# Cardioprotective Effects of Genistin in Rat Myocardial Ischemia-Reperfusion Injury Studies by Regulation of P2X7/NF-*κ*B Pathway

**DOI:** 10.1155/2016/5381290

**Published:** 2016-03-20

**Authors:** Meng Gu, Ai-bin Zheng, Jing Jin, Yue Cui, Ning Zhang, Zhi-ping Che, Yan Wang, Jie Zhan, Wen-juan Tu

**Affiliations:** ^1^Department of Clinical Laboratory, Changzhou Children's Hospital, Changzhou, Jiangsu 213001, China; ^2^Department of Neonatal, Changzhou Children's Hospital, Changzhou, Jiangsu 213001, China

## Abstract

The present study aimed to assess the effects and mechanisms of genistin in the rat model of myocardial ischemia reperfusion injury. The rat hearts were exposed to the left anterior descending coronary artery (LAD) ligation for 30 min followed by 1 h of reperfusion. In the rat of myocardial ischemia/reperfusion (MI/R), it was found that genistin pretreatment reduced myocardial infarct size, improved the heart rate, and decreased creatine kinase (CK) and lactate dehydrogenase (LDH) levels in coronary flow. This pretreatment also increased catalase (CAT), superoxide dismutase (SOD) activities but decreased glutathione (GSH), malondialdehyde (MDA) levels. Furthermore, we determined that genistin can ameliorate the impaired mitochondrial morphology and oxidation system; interleukin-6 (IL-6), interleukin-8 (IL-8), interleukin-10 (IL-10), and tumor necrosis factor-*α* (TNF-*α*) levels were also recovered. Besides, related-proteins of nuclear factor kappa-B (NF-*κ*B) signal pathway activated by P2X7 were investigated to determine the molecular mechanism of genistin and their expressions were measured by western blot. These results presented here demonstrated that genistin enhanced the protective effect on the rats with myocardial ischemia reperfusion injury. Therefore, the cardioprotective effects of genistin may rely on its antioxidant and anti-inflammatory activities via suppression of P2X7/NF-*κ*B pathways.

## 1. Introduction

The myocardial injury in ischemic heart diseases is mainly associated with ischemia/reperfusion (I/R) injury. It is a principal cause of death and disability all over the world [1]. Previous studies showed that promptly resuming the blood supply in the ischemic tissue is the most effective way of treating acute myocardial infarction and reperfusion induced oxidative stress plays a critical role in this pathology [2]. Thus, many studies exerted considerable efforts to elucidate the mechanism of cardioprotection. However, reperfusion itself after even brief duration of ischemia causes other irreversible myocardial damages, which is called myocardial ischemia reperfusion (IR) injuries. It is often associated with microvascular dysfunction including impaired endothelial-dependent dilation in arterioles, excess fluid filtration, and leukocyte plugging of capillaries [3]. As a complicated pathological process, the mechanism of MI/R is still largely unclear.

Recently, P2X7, an ion channel protein, has been reported to play a key role in the immune system and oxidative stress. The P2X7 receptor is expressed in very different tissues, and its activation can trigger multiple cellular responses. It has been reported that reactive oxygen species (ROS) overproduction is responsible for P2X7 receptor activation [4]. In addition, P2X7 has been shown to increase NADPH oxidase activity. It was developed as a potential new target for the treatment of inflammatory diseases of which the specific P2X7 receptor antagonist and P2X7 receptor knockout animal model has been used extensively in some studies [5]. The activation of P2X7/NF-*κ*B signal pathway stimulated the expression of upstream gene IKK, as well as the p65 subunit of NF-*κ*B, both of which intervened in inflammatory responses and oxidative stress. Taking together, these evidences indicate that P2X7/NF-*κ*B signal may play a significant role in lung regulatory pathways.

Flavonoids, which are a group of naturally occurring secondary metabolites that are widely distributed in the plant kingdom, possess unique antioxidant activities and other pharmacological effects that may be relevant in protecting the heart from I/R injury [6]. These flavonoids prevent oxidant production through xanthine oxidase inhibition and transition metal chelation, which could reduce oxidative stress from attacking cellular targets, block oxidative reactions, and enhance cellular antioxidant capacity by minimizing the effects of oxidants [7]. Flavonoids have also demonstrated that they could play the effects of anti-inflammatory and antiplatelet aggregation by controlling correlative enzymes and signal pathways. So they could ultimately reduce oxidant production and enhance the reestablishment of blood flow in the ischemic site. In addition, the recently research has focused on the mitochondrial pathway, through which certain flavonoids can play a target of the cardioprotection [8]. Finally, flavonoids also exhibit vasodilator effects via various mechanisms, one of which may be interacted with ion channels [9]. These diverse effects of flavonoids raise their utility as potential therapeutic intervention tools to protect I/R injury.

Genistin, a flavonoid which is abundant in the annual plant of Fabaceae family especially* Glycine max* (L.) MERR, has been reported to possess various therapeutic effects, including anti-inflammatory and anticancer activities [10, 11]. It can prevent oxidant damage and cell apoptosis by several mechanisms [12]. Furthermore, genistin has been shown to be a strong antioxidative agent [13]. Meanwhile, genistin, the aglycone of genistin, could also inhibit lipid peroxidation and reduce infarct size and apoptosis of myocytes, suggesting the effect on myocardial ischemia/reperfusion injury [14, 15]. Here, we explored the antioxidative and cardioprotective effects of pretreatment with genistin against IR injuries. The study presented here was undertaken to evaluate the protective effects of genistin on the I/R rats and to illustrate whether its cardioprotective effects associated with P2X7/NF-*κ*B pathway.

## 2. Materials and Methods

### 2.1. Materials

Genistin (pure: 95%) was provided by the National Institutes for the Control of Pharmaceutical and Biological Products (Beijing, China), and its purity was identified by high performance liquid chromatography (HPLC). Superoxide dismutase (SOD), malondialdehyde (MDA), and lactate dehydrogenase (LDH) kits were purchased from Nanjing Jiancheng Bioengineering Research Institute (Nanjing, China). Interleukin-6 (IL-6), interleukin-8 (IL-8), interleukin-10 (IL-10), and tumor necrosis factor-*α* (TNF-*α*) Elisa kits were obtained from R&D Systems Inc. (Minneapolis, MN, USA).

### 2.2. Animals

Sprague-Dawley rats (male, 250–300 g) were purchased from Shanghai Slac Laboratory Animal Ltd. (Shanghai, China). All animals were housed with free access to water and food with a 12 h light/dark cycle at the constant temperature (22 ± 2°C). Rats were acclimated for 7 days before any experimental procedures. The experiments were conducted in adherence with the National Institutes of Health Guidelines for the Use of Laboratory Animals.

### 2.3. Experimental Design

The I/R injury animal model was established by the left anterior descending (LAD) coronary artery ligation for 30 min followed by 1 h reperfusion. Briefly, after being anesthetized with a 30 mg/kg pentobarbital sodium intraperitoneally, rats were ventilated with a positive pressure respirator at a stroke volume of 12 mL/kg and a rate of 60 strokes per minute with 95% O_2_ and 5% CO_2_ throughout the experiment. The rat heart was exposed through a left thoracotomy. Then, the LAD was ligated 2-3 mm from its origin and loosened to simulate I/R rat model (ischemia for 30 min and reperfusion for 1 h). Rats were randomly apportioned in equal animals (*n* = 10) to five experimental groups: (1) sham group: rats were subjected to the entire surgical procedure but without the induction of I/R; (2) model group: I/R injury animal model was constructed by LAD ligation for 30 min, and then the LAD was allowed 1 h reperfusion; and (3) three genistin-treated groups: different doses (20, 40, and 60 mg/kg body weight, resp.) of genistin dissolved in 0.5% sodium carboxyl methyl cellulose (CMC-Na) solution were given intragastrically for 5 days before operation.

### 2.4. Evaluation of Myocardial Infarct Area

To further evaluate the myocardial infarct sizes, tetrazolium chloride (TTC) staining was adopted. After reperfusion, the isolated rat hearts were immediately washed in phosphate-buffered saline, frozen, stored at −20°C for 30 min, and then sectioned into 5 mm transverse slices. After incubation in 1% (0.01 g/mL) TTC at 37°C in PBS for 15 min, the heart slices were photographed with a digital camera to distinguish the red-stained viable tissues and the white-unstained necrotic tissues. Areas of infarct size were measured digitally using Image Pro Plus software.

### 2.5. Histopathologic Examination of Hearts

To investigate the effects of genistin on the protecting from the myocardial ischemia-reperfusion injury (MIRI) rats, hematoxylin-eosin staining (HE) was performed for hearts. A small piece of subendocardial myocardium from the root of the left ventricular papillary muscle was excised, rinsed with saline solution, fixed overnight in 4% fresh paraformaldehyde, and embedded in paraffin. Of 5 *μ*m sections were obtained, stained with HE, and performed of the section for observation of pathological changes in the heart tissues under a light microscopy and photomicrographs were taken.

### 2.6. Determination of Creatine Kinase (CK) and Lactate Dehydrogenase (LDH) in Serum

At the end of reperfusion, 2 mL of femoral vein blood was collected and centrifuged at 3,000 rpm for 20 min. Serum CK and LDH levels were analyzed by colorimetry according to the manufacturer's instructions. The activities of these enzymes were expressed in U/L.

### 2.7. Assay of Oxidative Stress

After perfusions, hearts were harvested and maintained at −70°C for later analysis. The frozen ventricles were crushed to a powder by liquid nitrogen-chilled tissue pulverizer. For tissue analysis, weighed amounts of the frozen tissues were homogenized in appropriate buffer using a microcentrifuge tube homogenizer.

SOD activity, MDA level, CAT activity, and GSH level were spectrophotometrically analyzed according to the instruction of assay kits.

### 2.8. Assay of Inflammation

TNF-*α*, IL-6, IL-8, and IL-10 were spectrophotometrically analyzed using ELISA following the manufacturer's instructions.

### 2.9. Western Blot of P2X7/NF-*κ*B Pathways

The myocardial tissues were removed and washed with PBS. Then the samples were cut into pieces and homogenized. Proteins were extracted with lysis buffer (RIPA with protease and phosphatase inhibitor) for 20 min on ice. The samples were loaded to 12% sodium dodecyl sulphate polyacrylamide gel electrophoresis (SDS-PAGE) gels and transferred to polyvinylidene fluoride (PVDF) membranes. The membrane was blocked with blocking reagent (20 mM Tris (pH 7.4), 125 mM NaCl, and 0.2% (vol/vol) Tween 20, 4% (wt/vol) nonfat dry milk, and 0.1% (wt/vol) sodium azide) for 2 h at room temperature and then incubated with primary antibodies diluted 1 : 1000 overnight at 4°C. After incubation with the corresponding horseradish peroxidase-conjugated secondary antibodies for 2 h, the bounds were detected using the SuperSignal West Pico Chemiluminescent Substrate and quantified using the Quantity One System (Bio-Rad, Hercules, CA, USA).

### 2.10. Statistical Analysis

The results were expressed as mean ± SEM. All data were processed with SPSS 11.0 statistical package for Windows version. The comparison of data from multigroup was analyzed using one-way ANOVA, followed by Student-Newman-Keuls's post hoc test. *P* values < 0.05 or < 0.01 were considered significant or highly significant, respectively.

## 3. Results

### 3.1. Effect of Genistin on the Myocardial Infarction Size of Hearts

**Figure 1:**
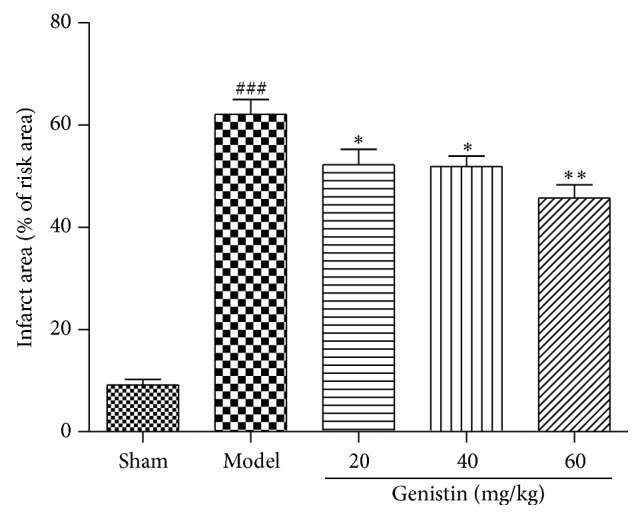
Effect of genistin on myocardial damage in rats subjected to MI/R. TTC staining to assess the extent of myocardial necrosis. Bars represent the percent of ischemic area at risk in hearts. Values are means ± SEM, *n* = 6 per group. ^###^ *P* < 0.001 compared with the sham group; ^*∗*^ *P* < 0.05, ^*∗∗*^ *P* < 0.01 compared with the model group.

To evaluate the direct effect of genistin on myocardial I/R injury, TTC staining was used to analyze the infarct area (Figure 1). Myocardial infarct was significantly increased in IR group compared with the sham group. In contrast, this effect was markedly diminished by pretreatment with genistin, particularly at the high dose.

### 3.2. Genistin Impaired Myocardial Structure Turbulence Induced by I/R Injury

**Figure 2:**
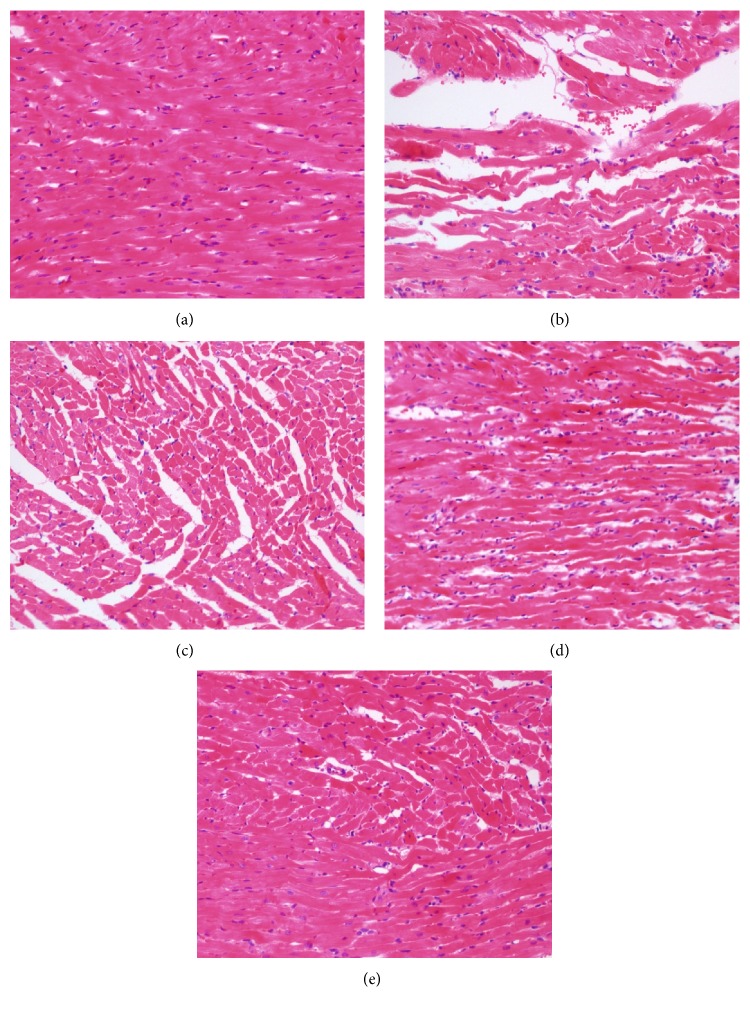
Histopathological changes in the myocardium following reperfusion (magnification, ×100). Sham-operated group (a); model group (b); genistin (20, 40, and 60 mg/kg) group (c, d, and e).

The changes in the morphological structures of myocardial tissues were evaluated by HE coloration. Optical micrographs of rat myocardial structures are shown in Figure 2. The myocardial membrane damage and infiltration of inflammatory cells were observed in the myocardial structures of I/R group as compared to those of sham control group. Moreover, compared with the I/R group, the group pretreated with genistin showed marked improvement evidenced by reduced degree of myonecrosis, edema, infiltration of inflammatory cells, and lesser vacuolar changes compared to the I/R group.

### 3.3. Genistin Reduces LDH, CK Release after I/R Injury in Rats

**Figure 3:**
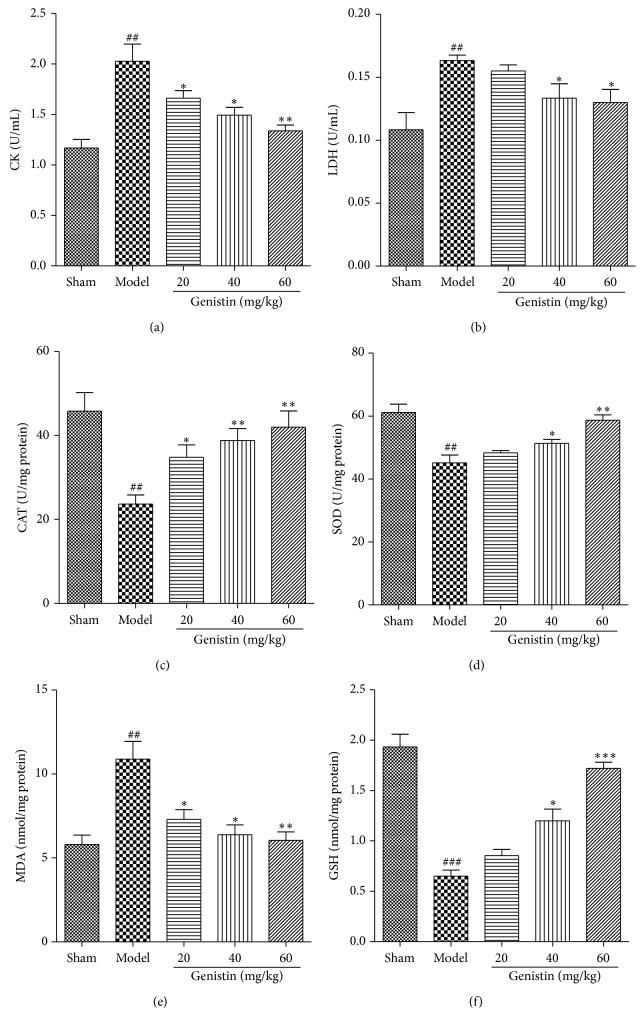
Effect of genistin treatment on serum CK (a) and LDH (b) levels in rats subjected to myocardial ischemia-reperfusion. And effect of genistin treatment on CAT activity (c), SOD activity (d), MDA level (e), and GSH level (f). Data represent the means ± SEM in each group (*n* = 6); ^##^ *P* < 0.01, ^###^ *P* < 0.001 compared with the sham group; ^*∗*^ *P* < 0.05, ^*∗∗*^ *P* < 0.01, and ^*∗∗∗*^ *P* < 0.001 compared with the model group.

As shown in Figure 3, levels of serum CK and LDH were remarkably increased in rats of I/R group compared with control group. After 1 h of reperfusion, preconditioning with genistin at dosages of 20–60 mg/kg significantly attenuated the release of LDH, CK in a dose-dependent manner compared with the I/R group.

### 3.4. Genistin Ameliorated Oxidative Stress of Myocardial Tissues Induced by I/R Injury

SOD activity, CAT activity, MDA level, and GSH are indicators of oxidative stress. These indicators were determined in myocardial tissues to identify the possible mechanisms underlying the cardioprotective effects of genistin. As illustrated in Figure 3, the result showed that the level of MDA was decreased and the activities of SOD and CAT were increased as well as an increased GSH level in a dose-dependent manner by genistin treatment in I/R.

### 3.5. The Expression of IL-6, IL-8, IL-10, and TNF-*α*

**Figure 4:**
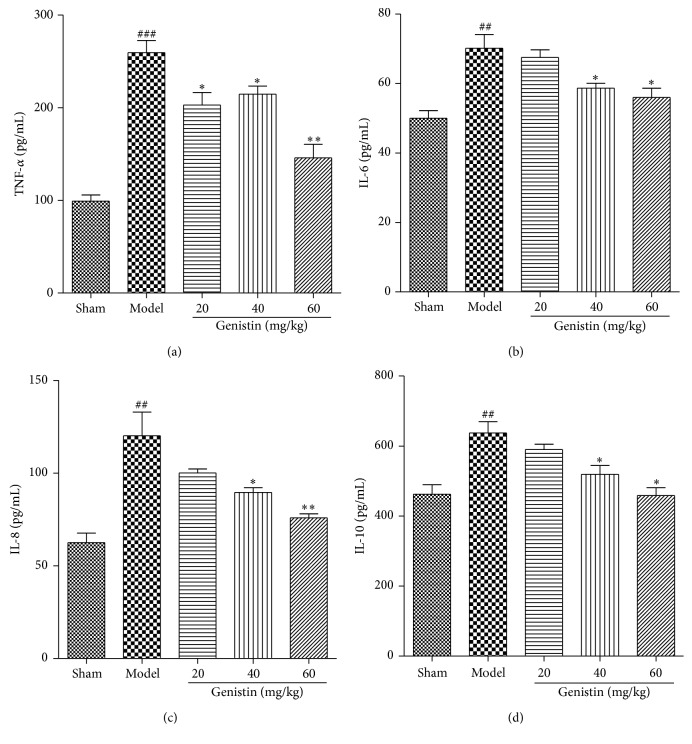
Effect of genistin on inflammatory cytokine production in the serum. The levels of TNF-*α*, IL-6, IL-8, and IL-10 were measured by ELISA kits. Data represent the means ± SEM in each group (*n* = 6); ^##^ *P* < 0.01, ^###^ *P* < 0.001 compared with the sham group; ^*∗*^ *P* < 0.05, ^*∗∗*^ *P* < 0.01 compared with the model group.

Figure 4 showed the change of serum IL-6, IL-8, IL-10, and TNF-*α* concentration of all the tested rats. In this study, we found that the plasma levels of IL-6, IL-8, IL-10, and TNF-*α* were significantly elevated following I/R injury compared with sham group. Furthermore, our results determined that pretreatment with genistin significantly attenuated the cytokines release. As expected, the treatment of genistin dose-dependently protected the rats of I/R injury. In the high dosage group, the levels of inflammatory cytokines were mostly close to the sham rats. Serum IL-6, IL-8, IL-10, and TNF-*α* concentrations decreased 21.3%, 36.9%, 28.1%, and 44.4%, respectively.

### 3.6. Effect of Genistin on P2X7/NF-*κ*B Pathway and Related Proteins

**Figure 5:**
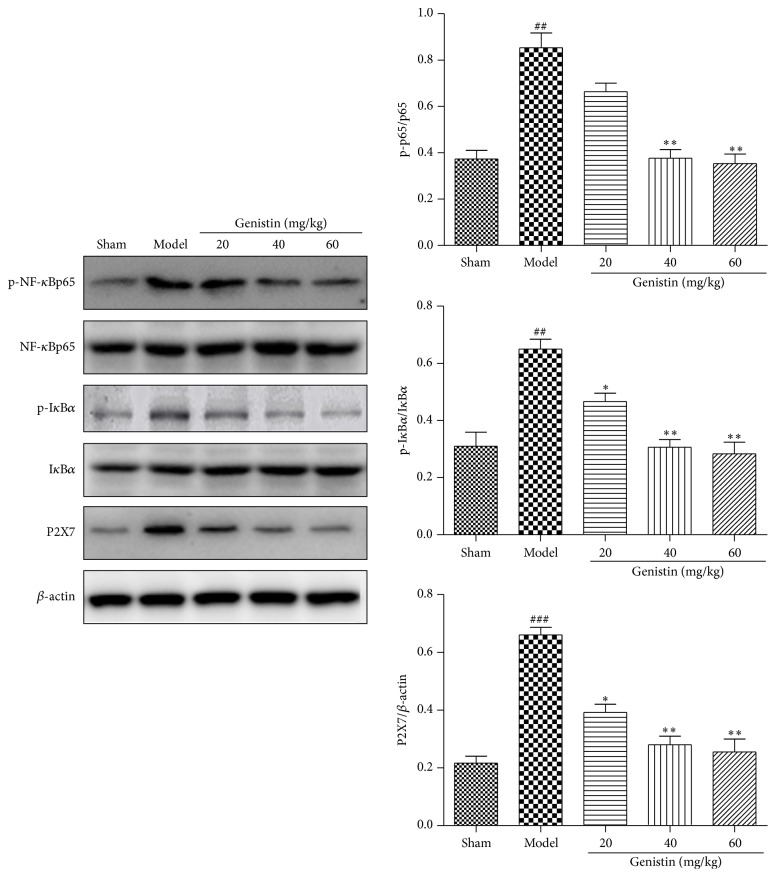
The protein levels of p-I*κ*B*α*, I*κ*B*α*, NF-*κ*B, p-NF-*κ*B, and P2X7 in rat myocardial tissue were detected by western blot. Data represent the means ± SEM in each group (*n* = 3); ^##^ *P* < 0.01, ^###^ *P* < 0.001 compared with the sham group; ^*∗*^ *P* < 0.05, ^*∗∗*^ *P* < 0.01 compared with the model group.

To further determine the underlying damage mechanism, the western blot was performed to investigate the activation of I*κ*B*α*, NF-*κ*Bp65, and their corresponding phosphorylated forms, as well as P2X7. As shown in Figure 5, it could be observed that the expression of p-I*κ*B*α*, p-NF-*κ*BP65, and P2X7 protein levels was significantly increased in model group compared with the sham group. Moreover, pretreatment with genistin (20, 40 and 60 mg/kg) prevented the expression of P2X7, p-I*κ*B*α*, and p-NF-*κ*B p65 compared with the model group.

## 4. Discussion

In the present study, we investigated for the first time the protective effects of genistin on myocardial ischemia-reperfusion injury. The results demonstrated that preconditioning with genistin remarkably improved the I/R-induced cardiac injury through inhibition inflammation and relieved the oxidative stress, whereas genistin affected the pathway of P2X7/NF-*κ*B. Moreover, the treatment of genistin, which reduced the myocardial infarct size, may work as a cardioprotective agent.

Nowadays, a number of epidemiological studies have reported that inflammatory lesions play a core role in the MI/R process. Cytokines, a heterogeneous group of proteins, have been associated with the inflammatory response in the progress of ischemia/reperfusion injury [16]. It has been shown that ischemia/reperfusion (I/R) increases the relative levels of various cytokines, such as TNF-*α*, IL-6, IL-8, and IL-10, in the myocardium. TNF-*α*, as a critical early mediator, plays a very crucial role in the genesis of a systemic inflammatory response [17]. In addition, it could stimulate the secretion of secondary cytokines, including the proinflammatory IL-6 and the anti-inflammatory IL-10 [18]. Here, we determined the serum levels of IL-6, IL-8, IL-10, and TNF-*α*. The levels of these cytokines in the I/R rats are in accordance with those presented in other investigations which showed that myocardium synthesizes and releases TNF-*α*, IL-6, IL-8, and IL-10 in response to I/R. Moreover, the genistin, the aglycone of genistin, mainly derived from soybean, has been demonstrated to be able to play many protective effects on the cardiovascular system. These beneficial actions of genistin showed the protected against myocardial I/R injury [14]. However, genistin preconditioning significantly reversed the response, suggesting the anti-inflammation properties of genistin were involved in its cardioprotective effect in the I/R rats.

To further clearly determine the relationship between inoxidizability and the cardioprotection of genistin, an experiment was carried out to examine whether genistin affected the changes in MDA and GSH levels, SOD and CAT activities induced by I/R. From the results, we found the I/R rats showed an increase in MDA production as well as a decrease in SOD level and GSH. MDA is considered to affect the generation and production of the ROS, which is caused by peroxidation of cell membrane lipids [19]. SOD, as one of the most significant intracellular antioxidant enzymes, could function as a ROS scavenger. GSH, a tripeptide composed of glutamate, exerts a critical role as antioxidant and neuromodulator in the central nervous system [20]. The imbalance between oxidation and antioxidation leads to the oxidation injury. In our study, genistin dose-dependently reduced the increased levels of MDA, LDH, and CK, especially at 60 mg/kg dose. Moreover, genistin increased the activity of the antioxidase SOD, compared with the I/R group. Taken these results together with the experiment data, it was suggested that the protective ability of genistin against ischemia/reperfusion injury in vivo was exerted by means of mediating reactive oxygen species. To further characterize the cardioprotective mechanism of genistin on MIRI rat, we evaluated the effects of genistin on the activation of the P2X7/NF-*κ*B signaling pathways. P2X7 can selectively target NF*κ*B-p65 and activation of P2X7 is required for the production and release of many inflammatory factors like IL-1*β*, IL-18, IL-6, and TNF-*α* [21]. Moreover, P2X7 participate in the regulation of oxidative stress [22]. In this study, we observed that the level of P2X7 was basically recovered to the normal level after genistin treatment at the 60-dose group. The levels of phosphorylation of NF-*κ*B P65 and I*κ*B*α* were markedly increased in the I/R group, and administration of genistin impairs phosphorylation of these molecules in a dose-dependent manner. The present results clearly demonstrate that genistin obviously regulated P2X7/NF-*κ*B pathway.

In conclusion, on the basis of present study findings from the hemodynamic, biochemical, and histopathological results, we confirmed that genistin, as an antioxidant and anti-inflammation agent, could attenuate the myocardial ischemia-reperfusion injury. The present results also clearly demonstrated the mechanism of genistin by regulating the P2X7/NF-*κ*B pathway to protect the MIRI rats. Our findings may advance the possible utility of genistin as an ideal agent for patients with I/R injury.
